# *catena*-Poly[[bis­(μ_2_-1,4,7,10,13,16-hexa­oxacyclo­octa­deca­ne)dipotassium]-μ_2_-iodido-(iodidocadmium)-di-μ_2_-iodido-(iodidocadmium)-μ_2_-iodido]

**DOI:** 10.1107/S1600536813002274

**Published:** 2013-01-31

**Authors:** K. Rajarajan, A. Pugazhenthi, M. NizamMohideen

**Affiliations:** a Department of Physics, Rajeswari Vedachalam Government Arts College, Chengalpet 603 001, India; b Research and Development Centre, Bharathiyar University, Coimbatore 641 046, India; c Department of Physics, The New College (Autonomous), Chennai 600 014, India

## Abstract

The reaction of CdCl_2_, 18-crown-6 and KI in water yields the title coordination polymer, [{K(C_12_H_24_O_6_)}_2_Cd_2_I_6_]_*n*_. The potassium ion lies approximately in the plane of the crown ether, coordinated by all six crown ether O atoms and also by an iodide anion bound to a cadmium atom. A C atom of the crown ether is disordered over two positions with site occupancies of 0.77 (2) and 0.23 (2). Two K(18-crown-6)^+^ units are linked by inversion symmetry, forming a [bis­(μ_2_-18-crown-6)dipottasium] system with approximately square-planar K_2_O_2_ units. Inversion symmetry also generates the Cd_2_I_6_ fragment and the polymeric system is extended along the *c* axis by the formation of K—I—Cd bridges.

## Related literature

For applications of polyiodides, see: Yang *et al.* (2011 ▶). For the properties of cadmium compounds, see: Ramesh *et al.* (2012 ▶). For related structures, see: Park *et al.* (2010 ▶); Guo *et al.* (2006 ▶); Kunz *et al.* (2009 ▶).
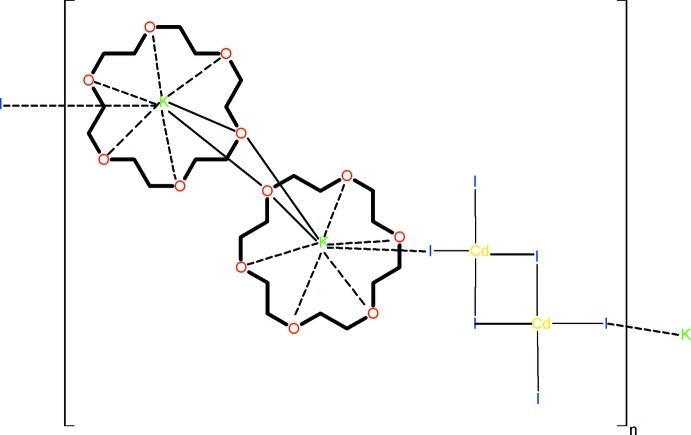


## Experimental

### 

#### Crystal data

[Cd_2_K_2_I_6_(C_12_H_24_O_6_)_2_]
*M* *_r_* = 796.51
Monoclinic, 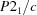

*a* = 10.627 (2) Å
*b* = 14.986 (2) Å
*c* = 15.190 (3) Å
β = 103.959 (1)°
*V* = 2347.7 (7) Å^3^
*Z* = 4
Mo *K*α radiation
μ = 5.07 mm^−1^
*T* = 293 K
0.20 × 0.15 × 0.10 mm

#### Data collection

Bruker Kappa APEXII CCD diffractometer
Absorption correction: multi-scan (*SADABS*; Sheldrick, 2004 ▶) *T*_min_ = 0.430, *T*_max_ = 0.631
21969 measured reflections
4129 independent reflections
3585 reflections with *I* > 2σ(*I*)
*R*_int_ = 0.025

#### Refinement

*R*[*F* ^2^ > 2σ(*F* ^2^)] = 0.028
*wR*(*F* ^2^) = 0.067
*S* = 1.03
4129 reflections
219 parameters
6 restraints
H-atom parameters constrained
Δρ_max_ = 1.53 e Å^−3^
Δρ_min_ = −1.16 e Å^−3^

### 

Data collection: *APEX2* (Bruker, 2004 ▶); cell refinement: *APEX2* and *SAINT* (Bruker, 2004 ▶); data reduction: *SAINT* and *XPREP* (Bruker, 2004 ▶); program(s) used to solve structure: *SHELXS97* (Sheldrick, 2008 ▶); program(s) used to refine structure: *SHELXL97* (Sheldrick, 2008 ▶); molecular graphics: *ORTEP-3 for Windows* (Farrugia, 2012 ▶) and *Mercury* (Macrae *et al.*, 2008 ▶); software used to prepare material for publication: *WinGX* (Farrugia, 2012 ▶) and *PLATON* (Spek, 2009 ▶).

## Supplementary Material

Crystal structure: contains datablock(s) global, I. DOI: 10.1107/S1600536813002274/sj5295sup1.cif

Structure factors: contains datablock(s) I. DOI: 10.1107/S1600536813002274/sj5295Isup2.hkl

Additional supplementary materials:  crystallographic information; 3D view; checkCIF report

## Figures and Tables

**Fig. 1.**
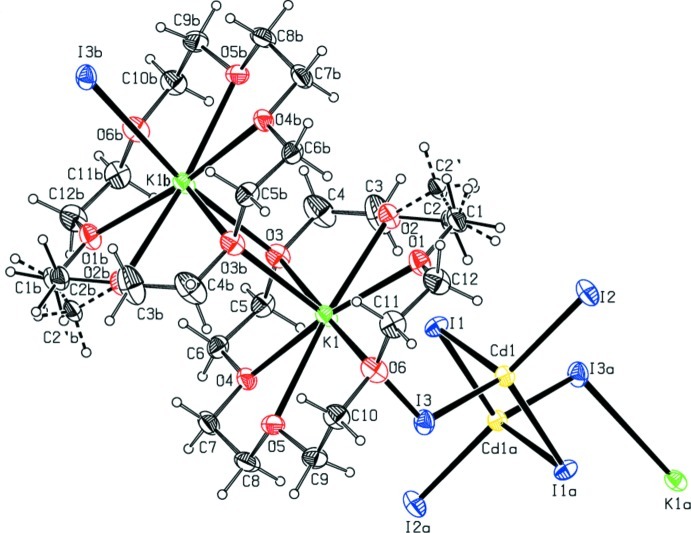
View of the title compound showing the atom-numbering scheme. Displacement ellipsoids are drawn at the 40% probability level. H atoms are presented as a small spheres of arbitrary radius.

**Fig. 2.**
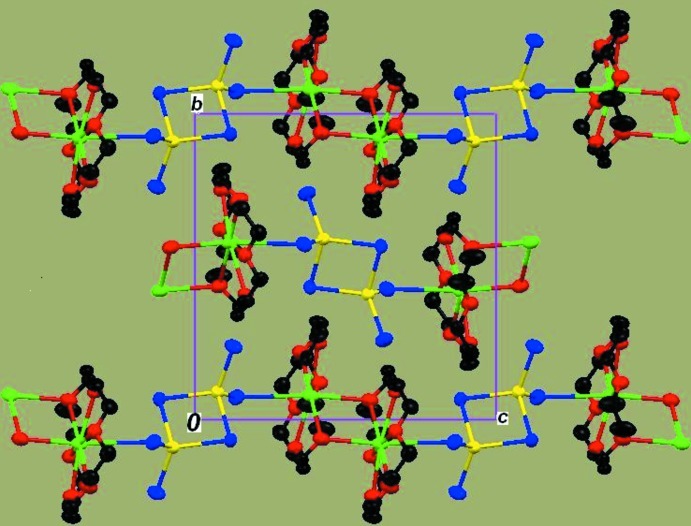
Molecular packing viewed along the a axis.

## supplementary crystallographic information

### Comment

There is a general interest in polyiodides as they are well known for having a significant influence on the redox chemistry in dye-sensitized solar cells (Yang *et al.*, 2011).

In a recent paper, we reported the synthesis and crystal structure of *catena*-Poly[ammonium (cadmium-tri-lthiocyanato- κ^4^*S*:*N*;κ^2^*N*:*S*)-1,4,10,13,16- hexaoxacyclooctadecane (1/1)] (Ramesh *et al.*, 2012). As part of our ongoing investigation of Cd and 18-crown-6 derivatives, we report here the synthesis and structure of the title compound.

The reaction of CdCl_2_ with 18-crown-6 and KI in de-ionized water yields the title coordination polymer [{K(C_12_H_24_O_6_)}_2_Cd_2_I_6_]_n_ (Fig.1). The carbon atom (C2) of the crown ether is disordered, as detectable from the large displacement parameters for the C atom and short C-O bond lengths. The disorder over two positions was modelled and the site occupancies refined to 0.77 (2) and 0.23 (2). The geometry was regularized by soft restraints.

The potassium ion lies close to the plane of the crown ether deviating by 0.1257 (2)Å from the mean plane of the six crown ether oxygen atoms (Kunz *et al.,* 2009). The K-O (K1, O1-O6) distances range from 2.686 (4) to 3.011 (4)Å and compare well to those of similar complexes (Kunz *et al.*, 2009). K1 is coordinated by all six oxygen atoms of the crown ether and also by the I3 iodide anion bound to the Cd(II) cation forming a K1—I3—Cd1 bridge. The mean Cd-I (Cd1, I1-I3) bond lengths [2.764 (2)Å] and angles around the Cd1 atom range from 95.0 (2)° to 115.5 (2)° and are similar to those reported for comparable complexes (Park *et al.,* 2010; Guo *et al.,* 2006).

Fig. 2. shows a view of the crystal structure down the *a* axis. The title compound is stabilized by van der Waals forces only, and no classical intra- or intermolecular hydrogen bonds are found.

### Experimental

A mixture of 18-crown-6 (C_12_H_24_O_6_), CdCl_2_ and KI (molar ratio 1:1:3) was thoroughly dissolved in de-ionized water and stirred for 4 h to obtain a homogeneous mixture. Colorless single crystals were obtained after the filtrate had been allowed to stand at room temperature for two weeks.

### Refinement

The C2 atom of the crown ether is disordered over two positions with refined occupancies of 0.77 (2) and 0.23 (2). The corresponding bond distances involving the disordered atoms were restrained to be equal. Carbon H atoms were placed geometrically (C—H = 0.97 Å) and treated as riding with *U*_iso_(H) = 1.2U_eq_(C).

### Crystal data

**Table:**

[Cd_2_K_2_I_6_(C_12_H_24_O_6_)_2_]	*F*(000) = 1480
*M**_r_* = 796.51	*D*_x_ = 2.254 Mg m^−^^3^
Monoclinic, *P*2_1_/*c*	Mo *K*α radiation, λ = 0.71073 Å
Hall symbol: -P 2ybc	Cell parameters from 5995 reflections
*a* = 10.627 (2) Å	θ = 2.4–31.1°
*b* = 14.986 (2) Å	µ = 5.07 mm^−^^1^
*c* = 15.190 (3) Å	*T* = 293 K
β = 103.959 (1)°	Block, colorless
*V* = 2347.7 (7) Å^3^	0.20 × 0.15 × 0.10 mm
*Z* = 4	

### Data collection

**Table:**

Bruker Kappa APEXII CCD diffractometer	4129 independent reflections
Radiation source: fine-focus sealed tube	3585 reflections with *I* > 2σ(*I*)
Graphite monochromator	*R*_int_ = 0.025
ω and φ scans	θ_max_ = 25.0°, θ_min_ = 2.4°
Absorption correction: multi-scan (*SADABS*; Sheldrick, 2004)	*h* = −12→12
*T*_min_ = 0.430, *T*_max_ = 0.631	*k* = −17→17
21969 measured reflections	*l* = −18→17

### Refinement

**Table:**

Refinement on *F*^2^	Secondary atom site location: difference Fourier map
Least-squares matrix: full	Hydrogen site location: inferred from neighbouring sites
*R*[*F*^2^ > 2σ(*F*^2^)] = 0.028	H-atom parameters constrained
*wR*(*F*^2^) = 0.067	*w* = 1/[σ^2^(*F*_o_^2^) + (0.023*P*)^2^ + 7.1196*P*] where *P* = (*F*_o_^2^ + 2*F*_c_^2^)/3
*S* = 1.03	(Δ/σ)_max_ < 0.001
4129 reflections	Δρ_max_ = 1.53 e Å^−^^3^
219 parameters	Δρ_min_ = −1.16 e Å^−^^3^
6 restraints	Extinction correction: *SHELXL*, Fc^*^=kFc[1+0.001xFc^2^λ^3^/sin(2θ)]^-1/4^
Primary atom site location: structure-invariant direct methods	Extinction coefficient: 0.00235 (8)

### Special details

**Table:**

Geometry. All e.s.d.'s (except the e.s.d. in the dihedral angle between two l.s. planes) are estimated using the full covariance matrix. The cell e.s.d.'s are taken into account individually in the estimation of e.s.d.'s in distances, angles and torsion angles; correlations between e.s.d.'s in cell parameters are only used when they are defined by crystal symmetry. An approximate (isotropic) treatment of cell e.s.d.'s is used for estimating e.s.d.'s involving l.s. planes.
Refinement. Refinement of *F*^2^ against ALL reflections. The weighted *R*-factor *wR* and goodness of fit *S* are based on *F*^2^, conventional *R*-factors *R* are based on *F*, with *F* set to zero for negative *F*^2^. The threshold expression of *F*^2^ > σ(*F*^2^) is used only for calculating *R*-factors(gt) *etc*. and is not relevant to the choice of reflections for refinement. *R*-factors based on *F*^2^ are statistically about twice as large as those based on *F*, and *R*- factors based on ALL data will be even larger.

### Fractional atomic coordinates and isotropic or equivalent isotropic displacement parameters (Å^2^)

**Table:**

	*x*	*y*	*z*	*U*_iso_*/*U*_eq_	Occ. (<1)
C1	0.3249 (6)	0.2719 (4)	0.4090 (5)	0.0679 (16)	
H1A	0.3011	0.3261	0.3740	0.081*	0.772 (18)
H1B	0.3200	0.2840	0.4708	0.081*	0.772 (18)
H1C	0.2860	0.2977	0.3501	0.081*	0.228 (18)
H1D	0.3298	0.3192	0.4533	0.081*	0.228 (18)
C2	0.2332 (7)	0.2020 (6)	0.3714 (8)	0.060 (2)	0.772 (18)
H2A	0.1481	0.2182	0.3790	0.072*	0.772 (18)
H2B	0.2275	0.1966	0.3070	0.072*	0.772 (18)
C2'	0.238 (3)	0.2087 (18)	0.426 (3)	0.055 (5)	0.228 (18)
H2'1	0.1528	0.2214	0.3879	0.066*	0.228 (18)
H2'2	0.2320	0.2155	0.4889	0.066*	0.228 (18)
C3	0.1840 (7)	0.0503 (5)	0.3846 (7)	0.110 (3)	
H3A	0.1013	0.0665	0.3965	0.131*	
H3B	0.1705	0.0449	0.3193	0.131*	
C4	0.2172 (7)	−0.0310 (5)	0.4214 (7)	0.104 (3)	
H4A	0.1537	−0.0738	0.3898	0.125*	
H4B	0.2110	−0.0294	0.4840	0.125*	
C5	0.3433 (6)	−0.1295 (4)	0.3525 (4)	0.0606 (14)	
H5A	0.3073	−0.1049	0.2927	0.073*	
H5B	0.2900	−0.1796	0.3614	0.073*	
C6	0.4776 (6)	−0.1597 (3)	0.3595 (4)	0.0577 (14)	
H6A	0.5175	−0.1768	0.4216	0.069*	
H6B	0.4772	−0.2115	0.3210	0.069*	
C7	0.6773 (6)	−0.1139 (4)	0.3315 (4)	0.0660 (16)	
H7A	0.6775	−0.1703	0.3001	0.079*	
H7B	0.7289	−0.1208	0.3932	0.079*	
C8	0.7332 (6)	−0.0427 (4)	0.2846 (4)	0.0678 (16)	
H8A	0.8191	−0.0600	0.2795	0.081*	
H8B	0.6792	−0.0342	0.2238	0.081*	
C9	0.7834 (6)	0.1110 (4)	0.2893 (4)	0.0642 (15)	
H9A	0.7216	0.1224	0.2321	0.077*	
H9B	0.8665	0.0970	0.2767	0.077*	
C10	0.7961 (5)	0.1915 (4)	0.3483 (5)	0.0684 (16)	
H10A	0.8492	0.1779	0.4082	0.082*	
H10B	0.8373	0.2394	0.3226	0.082*	
C11	0.6678 (6)	0.2865 (4)	0.4144 (5)	0.0721 (17)	
H11A	0.7194	0.3358	0.4010	0.087*	
H11B	0.7072	0.2660	0.4754	0.087*	
C12	0.5374 (6)	0.3184 (4)	0.4112 (5)	0.0760 (18)	
H12A	0.5394	0.3550	0.4642	0.091*	
H12B	0.5081	0.3555	0.3579	0.091*	
O1	0.4507 (4)	0.2494 (2)	0.4086 (3)	0.0636 (10)	
O2	0.2691 (3)	0.1203 (2)	0.4130 (3)	0.0595 (9)	
O3	0.3430 (4)	−0.0632 (2)	0.4197 (3)	0.0616 (10)	
O4	0.5494 (4)	−0.0901 (2)	0.3323 (2)	0.0539 (9)	
O5	0.7407 (4)	0.0380 (2)	0.3341 (2)	0.0568 (9)	
O6	0.6704 (4)	0.2180 (3)	0.3542 (3)	0.0680 (11)	
K1	0.51194 (11)	0.07876 (7)	0.38443 (8)	0.0520 (3)	
Cd1	0.10508 (3)	0.09039 (2)	0.07415 (2)	0.04486 (11)	
I1	−0.01908 (3)	−0.06719 (2)	0.11826 (2)	0.05110 (11)	
I2	−0.00034 (4)	0.23919 (3)	0.12810 (3)	0.06725 (14)	
I3	0.36601 (3)	0.07316 (3)	0.13778 (3)	0.05971 (12)	

### Atomic displacement parameters (Å^2^)

**Table:**

	*U*^11^	*U*^22^	*U*^33^	*U*^12^	*U*^13^	*U*^23^
C1	0.070 (4)	0.049 (3)	0.089 (4)	0.022 (3)	0.030 (3)	0.009 (3)
C2	0.050 (4)	0.066 (5)	0.059 (6)	0.019 (3)	0.004 (4)	0.005 (5)
C2'	0.048 (8)	0.060 (9)	0.058 (10)	0.024 (8)	0.012 (10)	0.002 (10)
C3	0.045 (4)	0.076 (5)	0.198 (10)	−0.006 (4)	0.009 (5)	0.005 (6)
C4	0.063 (4)	0.074 (5)	0.186 (9)	−0.012 (4)	0.052 (5)	−0.010 (5)
C5	0.071 (4)	0.050 (3)	0.059 (3)	−0.012 (3)	0.013 (3)	−0.002 (3)
C6	0.082 (4)	0.038 (3)	0.055 (3)	0.000 (3)	0.019 (3)	0.000 (2)
C7	0.069 (4)	0.049 (3)	0.085 (4)	0.018 (3)	0.028 (3)	−0.001 (3)
C8	0.072 (4)	0.065 (4)	0.076 (4)	0.011 (3)	0.038 (3)	−0.011 (3)
C9	0.053 (3)	0.076 (4)	0.070 (4)	−0.003 (3)	0.028 (3)	0.004 (3)
C10	0.050 (3)	0.070 (4)	0.089 (4)	−0.011 (3)	0.024 (3)	−0.002 (3)
C11	0.068 (4)	0.065 (4)	0.079 (4)	−0.010 (3)	0.009 (3)	−0.011 (3)
C12	0.086 (5)	0.046 (3)	0.100 (5)	−0.010 (3)	0.031 (4)	−0.013 (3)
O1	0.058 (2)	0.045 (2)	0.088 (3)	0.0044 (18)	0.018 (2)	−0.0001 (19)
O2	0.049 (2)	0.051 (2)	0.078 (3)	0.0035 (17)	0.0144 (19)	0.0012 (18)
O3	0.060 (2)	0.049 (2)	0.078 (3)	−0.0065 (18)	0.022 (2)	−0.0076 (18)
O4	0.061 (2)	0.0392 (18)	0.065 (2)	0.0083 (16)	0.0214 (18)	0.0028 (16)
O5	0.062 (2)	0.059 (2)	0.056 (2)	0.0023 (18)	0.0263 (18)	−0.0008 (17)
O6	0.058 (2)	0.058 (2)	0.092 (3)	−0.0112 (19)	0.024 (2)	−0.013 (2)
K1	0.0499 (6)	0.0392 (6)	0.0724 (8)	−0.0007 (5)	0.0256 (6)	−0.0059 (5)
Cd1	0.0438 (2)	0.0415 (2)	0.0481 (2)	−0.00042 (15)	0.00872 (15)	−0.00202 (15)
I1	0.0600 (2)	0.0530 (2)	0.04247 (19)	−0.01331 (16)	0.01657 (15)	0.00179 (14)
I2	0.0590 (2)	0.0554 (2)	0.0825 (3)	0.01439 (18)	0.0077 (2)	−0.01480 (19)
I3	0.0436 (2)	0.0646 (2)	0.0658 (2)	0.00437 (16)	0.00337 (16)	0.00944 (17)

### Geometric parameters (Å, º)

**Table:**

C1—O1	1.380 (7)	C8—O5	1.416 (6)
C1—C2'	1.39 (3)	C8—H8A	0.9700
C1—C2	1.451 (11)	C8—H8B	0.9700
C1—H1A	0.9700	C9—O5	1.419 (7)
C1—H1B	0.9700	C9—C10	1.489 (8)
C1—H1C	0.9700	C9—H9A	0.9700
C1—H1D	0.9700	C9—H9B	0.9700
C2—O2	1.388 (9)	C10—O6	1.417 (7)
C2—K1	3.456 (8)	C10—H10A	0.9700
C2—H2A	0.9700	C10—H10B	0.9700
C2—H2B	0.9700	C11—O6	1.379 (7)
C2'—O2	1.39 (3)	C11—C12	1.455 (8)
C2'—H2'1	0.9700	C11—K1	3.504 (6)
C2'—H2'2	0.9700	C11—H11A	0.9700
C3—C4	1.352 (10)	C11—H11B	0.9700
C3—O2	1.383 (8)	C12—O1	1.380 (7)
C3—H3A	0.9700	C12—H12A	0.9700
C3—H3B	0.9700	C12—H12B	0.9700
C4—O3	1.427 (8)	O1—K1	2.686 (4)
C4—H4A	0.9700	O2—K1	2.788 (4)
C4—H4B	0.9700	O3—K1	2.916 (4)
C5—O3	1.424 (6)	O3—K1^i^	3.011 (4)
C5—C6	1.477 (8)	O4—K1	2.709 (3)
C5—H5A	0.9700	O5—K1	2.786 (4)
C5—H5B	0.9700	O6—K1	2.788 (4)
C6—O4	1.413 (6)	K1—O3^i^	3.011 (4)
C6—H6A	0.9700	Cd1—I2	2.7097 (6)
C6—H6B	0.9700	Cd1—I3	2.7197 (7)
C7—O4	1.408 (6)	Cd1—I1	2.8624 (6)
C7—C8	1.485 (8)	Cd1—I1^ii^	2.8652 (7)
C7—H7A	0.9700	I1—Cd1^ii^	2.8652 (7)
C7—H7B	0.9700		
			
O1—C1—C2'	121.5 (11)	C11—C12—H12B	109.2
O1—C1—C2	112.4 (5)	H12A—C12—H12B	107.9
O1—C1—H1A	109.1	C12—O1—C1	117.3 (4)
C2'—C1—H1A	125.1	C12—O1—K1	122.4 (3)
C2—C1—H1A	109.1	C1—O1—K1	120.0 (3)
O1—C1—H1B	109.1	C3—O2—C2	116.1 (6)
C2'—C1—H1B	75.5	C3—O2—C2'	127.2 (12)
C2—C1—H1B	109.1	C3—O2—K1	110.0 (4)
H1A—C1—H1B	107.9	C2—O2—K1	106.8 (4)
O1—C1—H1C	107.0	C2'—O2—K1	119.8 (12)
C2'—C1—H1C	107.0	C5—O3—C4	114.3 (5)
C2—C1—H1C	80.2	C5—O3—K1	105.6 (3)
H1B—C1—H1C	135.0	C4—O3—K1	112.2 (4)
O1—C1—H1D	106.9	C5—O3—K1^i^	124.4 (3)
C2'—C1—H1D	106.9	C4—O3—K1^i^	105.5 (5)
C2—C1—H1D	136.0	K1—O3—K1^i^	92.68 (11)
H1A—C1—H1D	74.4	C7—O4—C6	113.9 (4)
H1C—C1—H1D	106.7	C7—O4—K1	116.7 (3)
O2—C2—C1	112.0 (6)	C6—O4—K1	118.5 (3)
O2—C2—K1	50.6 (3)	C8—O5—C9	112.8 (4)
C1—C2—K1	83.0 (4)	C8—O5—K1	113.1 (3)
O2—C2—H2A	109.2	C9—O5—K1	111.4 (3)
C1—C2—H2A	109.2	C11—O6—C10	114.9 (5)
K1—C2—H2A	159.8	C11—O6—K1	109.8 (3)
O2—C2—H2B	109.2	C10—O6—K1	114.6 (3)
C1—C2—H2B	109.2	O1—K1—O4	170.67 (13)
K1—C2—H2B	81.8	O1—K1—O5	120.16 (12)
H2A—C2—H2B	107.9	O4—K1—O5	61.03 (11)
C1—C2'—O2	115 (2)	O1—K1—O6	59.05 (12)
C1—C2'—H2'1	108.5	O4—K1—O6	120.91 (12)
O2—C2'—H2'1	108.5	O5—K1—O6	61.14 (11)
C1—C2'—H2'2	108.5	O1—K1—O2	60.16 (11)
O2—C2'—H2'2	108.5	O4—K1—O2	117.43 (12)
H2'1—C2'—H2'2	107.5	O5—K1—O2	173.22 (12)
C4—C3—O2	117.9 (7)	O6—K1—O2	118.49 (12)
C4—C3—K1	87.1 (4)	O1—K1—O3	119.29 (12)
O2—C3—K1	48.2 (3)	O4—K1—O3	60.85 (11)
C4—C3—H3A	107.8	O5—K1—O3	120.48 (11)
O2—C3—H3A	107.8	O6—K1—O3	178.21 (12)
K1—C3—H3A	156.0	O2—K1—O3	60.01 (11)
C4—C3—H3B	107.8	O1—K1—O3^i^	91.24 (12)
O2—C3—H3B	107.8	O4—K1—O3^i^	98.07 (11)
K1—C3—H3B	84.9	O5—K1—O3^i^	88.95 (11)
H3A—C3—H3B	107.2	O6—K1—O3^i^	91.99 (12)
C3—C4—O3	116.7 (7)	O2—K1—O3^i^	97.83 (11)
C3—C4—H4A	108.1	O3—K1—O3^i^	87.32 (11)
O3—C4—H4A	108.1	O1—K1—C2	42.81 (17)
C3—C4—H4B	108.1	O4—K1—C2	131.82 (18)
O3—C4—H4B	108.1	O5—K1—C2	153.38 (19)
H4A—C4—H4B	107.3	O6—K1—C2	97.71 (17)
O3—C5—C6	109.4 (4)	O2—K1—C2	22.61 (18)
O3—C5—H5A	109.8	O3—K1—C2	80.96 (17)
C6—C5—H5A	109.8	O3^i^—K1—C2	109.0 (2)
O3—C5—H5B	109.8	O1—K1—C11	42.17 (14)
C6—C5—H5B	109.8	O4—K1—C11	140.31 (14)
H5A—C5—H5B	108.2	O5—K1—C11	79.29 (13)
O4—C6—C5	109.5 (4)	O6—K1—C11	21.74 (13)
O4—C6—H6A	109.8	O2—K1—C11	101.99 (13)
C5—C6—H6A	109.8	O3—K1—C11	156.50 (14)
O4—C6—H6B	109.8	O3^i^—K1—C11	80.03 (13)
C5—C6—H6B	109.8	C2—K1—C11	84.52 (18)
H6A—C6—H6B	108.2	O1—K1—C3	80.91 (15)
O4—C7—C8	108.6 (5)	O4—K1—C3	95.91 (16)
O4—C7—H7A	110.0	O5—K1—C3	154.87 (17)
C8—C7—H7A	110.0	O6—K1—C3	136.86 (16)
O4—C7—H7B	110.0	O2—K1—C3	21.72 (16)
C8—C7—H7B	110.0	O3—K1—C3	41.94 (15)
H7A—C7—H7B	108.3	O3^i^—K1—C3	105.17 (19)
O5—C8—C7	109.7 (5)	C2—K1—C3	39.42 (19)
O5—C8—H8A	109.7	C11—K1—C3	123.07 (17)
C7—C8—H8A	109.7	O1—K1—I3	95.98 (9)
O5—C8—H8B	109.7	O4—K1—I3	74.79 (8)
C7—C8—H8B	109.7	O5—K1—I3	84.10 (8)
H8A—C8—H8B	108.2	O6—K1—I3	88.43 (9)
O5—C9—C10	109.2 (5)	O2—K1—I3	89.13 (9)
O5—C9—H9A	109.8	O3—K1—I3	92.49 (9)
C10—C9—H9A	109.8	O3^i^—K1—I3	171.82 (8)
O5—C9—H9B	109.8	C2—K1—I3	79.04 (19)
C10—C9—H9B	109.8	C11—K1—I3	102.81 (11)
H9A—C9—H9B	108.3	C3—K1—I3	79.86 (18)
O6—C10—C9	108.4 (5)	O1—K1—K1^i^	110.35 (10)
O6—C10—H10A	110.0	O4—K1—K1^i^	76.54 (8)
C9—C10—H10A	110.0	O5—K1—K1^i^	109.39 (9)
O6—C10—H10B	110.0	O6—K1—K1^i^	134.75 (10)
C9—C10—H10B	110.0	O2—K1—K1^i^	75.86 (8)
H10A—C10—H10B	108.4	O3—K1—K1^i^	44.54 (8)
O6—C11—C12	113.2 (5)	O3^i^—K1—K1^i^	42.78 (7)
O6—C11—K1	48.5 (3)	C2—K1—K1^i^	96.98 (18)
C12—C11—K1	82.6 (3)	C11—K1—K1^i^	120.13 (12)
O6—C11—H11A	108.9	C3—K1—K1^i^	71.22 (16)
C12—C11—H11A	108.9	I3—K1—K1^i^	136.43 (4)
K1—C11—H11A	157.3	I2—Cd1—I3	115.538 (18)
O6—C11—H11B	108.9	I2—Cd1—I1	111.13 (2)
C12—C11—H11B	108.9	I3—Cd1—I1	109.058 (17)
K1—C11—H11B	85.7	I2—Cd1—I1^ii^	110.792 (17)
H11A—C11—H11B	107.8	I3—Cd1—I1^ii^	113.455 (17)
O1—C12—C11	112.3 (5)	I1—Cd1—I1^ii^	94.994 (15)
O1—C12—H12A	109.2	Cd1—I1—Cd1^ii^	85.006 (15)
C11—C12—H12A	109.2	Cd1—I3—K1	120.06 (2)
O1—C12—H12B	109.2		
			
O1—C1—C2—O2	52.9 (10)	C3—O2—K1—O4	−8.3 (5)
C2'—C1—C2—O2	−60.7 (19)	C2—O2—K1—O4	−135.1 (5)
O1—C1—C2—K1	10.6 (5)	C2'—O2—K1—O4	−170.4 (18)
C2'—C1—C2—K1	−103 (2)	C3—O2—K1—O6	151.8 (5)
O1—C1—C2'—O2	−21 (3)	C2—O2—K1—O6	25.0 (5)
C2—C1—C2'—O2	63 (2)	C2'—O2—K1—O6	−10.3 (19)
O2—C3—C4—O3	−51.3 (13)	C3—O2—K1—O3	−29.3 (5)
K1—C3—C4—O3	−12.7 (8)	C2—O2—K1—O3	−156.1 (5)
O3—C5—C6—O4	−68.8 (6)	C2'—O2—K1—O3	168.6 (19)
O4—C7—C8—O5	63.5 (6)	C3—O2—K1—O3^i^	−111.6 (5)
O5—C9—C10—O6	−68.1 (6)	C2—O2—K1—O3^i^	121.6 (5)
O6—C11—C12—O1	44.6 (8)	C2'—O2—K1—O3^i^	86.2 (18)
K1—C11—C12—O1	6.1 (5)	C3—O2—K1—C2	126.8 (7)
C11—C12—O1—C1	177.6 (6)	C2'—O2—K1—C2	−35.3 (16)
C11—C12—O1—K1	−9.4 (8)	C3—O2—K1—C11	167.0 (5)
C2'—C1—O1—C12	−166 (2)	C2—O2—K1—C11	40.2 (5)
C2—C1—O1—C12	157.4 (7)	C2'—O2—K1—C11	4.8 (18)
C2'—C1—O1—K1	21 (2)	C2—O2—K1—C3	−126.8 (7)
C2—C1—O1—K1	−15.8 (8)	C2'—O2—K1—C3	−162.1 (19)
C4—C3—O2—C2	178.1 (9)	C3—O2—K1—I3	64.1 (5)
K1—C3—O2—C2	121.4 (7)	C2—O2—K1—I3	−62.7 (5)
C4—C3—O2—C2'	−143 (2)	C2'—O2—K1—I3	−98.1 (18)
K1—C3—O2—C2'	160 (2)	C3—O2—K1—K1^i^	−74.6 (5)
C4—C3—O2—K1	56.6 (10)	C2—O2—K1—K1^i^	158.6 (5)
C1—C2—O2—C3	177.0 (7)	C2'—O2—K1—K1^i^	123.2 (18)
K1—C2—O2—C3	−123.1 (7)	C5—O3—K1—O1	143.2 (3)
C1—C2—O2—C2'	58 (2)	C4—O3—K1—O1	18.0 (5)
K1—C2—O2—C2'	118 (2)	K1^i^—O3—K1—O1	−89.92 (14)
C1—C2—O2—K1	−59.9 (8)	C5—O3—K1—O4	−26.1 (3)
C1—C2'—O2—C3	−148.2 (16)	C4—O3—K1—O4	−151.3 (5)
C1—C2'—O2—C2	−65 (3)	K1^i^—O3—K1—O4	100.78 (13)
C1—C2'—O2—K1	11 (3)	C5—O3—K1—O5	−39.7 (4)
C6—C5—O3—C4	−178.0 (5)	C4—O3—K1—O5	−164.9 (5)
C6—C5—O3—K1	58.1 (5)	K1^i^—O3—K1—O5	87.20 (13)
C6—C5—O3—K1^i^	−46.3 (5)	C5—O3—K1—O2	132.5 (3)
C3—C4—O3—C5	−103.7 (8)	C4—O3—K1—O2	7.3 (5)
C3—C4—O3—K1	16.6 (10)	K1^i^—O3—K1—O2	−100.63 (13)
C3—C4—O3—K1^i^	116.1 (8)	C5—O3—K1—O3^i^	−126.9 (3)
C8—C7—O4—C6	168.6 (5)	C4—O3—K1—O3^i^	107.9 (5)
C8—C7—O4—K1	−47.6 (6)	K1^i^—O3—K1—O3^i^	0.0
C5—C6—O4—C7	−176.6 (5)	C5—O3—K1—C2	123.4 (4)
C5—C6—O4—K1	40.3 (5)	C4—O3—K1—C2	−1.8 (5)
C7—C8—O5—C9	−175.2 (5)	K1^i^—O3—K1—C2	−109.7 (2)
C7—C8—O5—K1	−47.7 (6)	C5—O3—K1—C11	175.9 (4)
C10—C9—O5—C8	−176.6 (5)	C4—O3—K1—C11	50.8 (6)
C10—C9—O5—K1	55.0 (5)	K1^i^—O3—K1—C11	−57.2 (4)
C12—C11—O6—C10	173.5 (5)	C5—O3—K1—C3	116.8 (4)
K1—C11—O6—C10	−130.9 (5)	C4—O3—K1—C3	−8.4 (5)
C12—C11—O6—K1	−55.5 (6)	K1^i^—O3—K1—C3	−116.4 (3)
C9—C10—O6—C11	173.6 (5)	C5—O3—K1—I3	44.9 (3)
C9—C10—O6—K1	45.0 (6)	C4—O3—K1—I3	−80.3 (5)
C12—O1—K1—O5	−10.5 (5)	K1^i^—O3—K1—I3	171.81 (8)
C1—O1—K1—O5	162.3 (4)	C5—O3—K1—K1^i^	−126.9 (3)
C12—O1—K1—O6	−12.6 (4)	C4—O3—K1—K1^i^	107.9 (5)
C1—O1—K1—O6	160.3 (4)	O2—C2—K1—O1	−133.4 (7)
C12—O1—K1—O2	177.3 (5)	C1—C2—K1—O1	−7.4 (4)
C1—O1—K1—O2	−9.8 (4)	O2—C2—K1—O4	57.3 (6)
C12—O1—K1—O3	166.6 (4)	C1—C2—K1—O4	−176.7 (4)
C1—O1—K1—O3	−20.5 (5)	O2—C2—K1—O5	166.8 (3)
C12—O1—K1—O3^i^	79.0 (5)	C1—C2—K1—O5	−67.1 (8)
C1—O1—K1—O3^i^	−108.2 (4)	O2—C2—K1—O6	−158.0 (5)
C12—O1—K1—C2	−163.9 (6)	C1—C2—K1—O6	−31.9 (5)
C1—O1—K1—C2	8.9 (5)	C1—C2—K1—O2	126.0 (9)
C12—O1—K1—C11	5.4 (4)	O2—C2—K1—O3	20.8 (5)
C1—O1—K1—C11	178.2 (5)	C1—C2—K1—O3	146.9 (5)
C12—O1—K1—C3	−175.8 (5)	O2—C2—K1—O3^i^	−63.2 (5)
C1—O1—K1—C3	−3.0 (4)	C1—C2—K1—O3^i^	62.9 (5)
C12—O1—K1—I3	−97.1 (5)	O2—C2—K1—C11	−140.6 (5)
C1—O1—K1—I3	75.7 (4)	C1—C2—K1—C11	−14.6 (5)
C12—O1—K1—K1^i^	118.2 (4)	O2—C2—K1—C3	27.8 (4)
C1—O1—K1—K1^i^	−69.0 (4)	C1—C2—K1—C3	153.9 (8)
C7—O4—K1—O5	16.9 (3)	O2—C2—K1—I3	115.1 (5)
C6—O4—K1—O5	159.0 (4)	C1—C2—K1—I3	−118.8 (5)
C7—O4—K1—O6	29.8 (4)	O2—C2—K1—K1^i^	−20.9 (5)
C6—O4—K1—O6	172.0 (3)	C1—C2—K1—K1^i^	105.1 (5)
C7—O4—K1—O2	−170.6 (3)	O6—C11—K1—O1	−134.5 (5)
C6—O4—K1—O2	−28.5 (4)	C12—C11—K1—O1	−4.3 (4)
C7—O4—K1—O3	−149.8 (4)	O6—C11—K1—O4	31.8 (5)
C6—O4—K1—O3	−7.6 (3)	C12—C11—K1—O4	161.9 (4)
C7—O4—K1—O3^i^	−67.4 (4)	O6—C11—K1—O5	31.6 (4)
C6—O4—K1—O3^i^	74.7 (4)	C12—C11—K1—O5	161.7 (4)
C7—O4—K1—C2	168.0 (4)	C12—C11—K1—O6	130.1 (6)
C6—O4—K1—C2	−49.8 (5)	O6—C11—K1—O2	−141.6 (4)
C7—O4—K1—C11	16.7 (5)	C12—C11—K1—O2	−11.5 (4)
C6—O4—K1—C11	158.8 (4)	O6—C11—K1—O3	−179.1 (3)
C7—O4—K1—C3	−173.7 (4)	C12—C11—K1—O3	−49.0 (6)
C6—O4—K1—C3	−31.5 (4)	O6—C11—K1—O3^i^	122.4 (4)
C7—O4—K1—I3	108.5 (4)	C12—C11—K1—O3^i^	−107.4 (4)
C6—O4—K1—I3	−109.3 (3)	O6—C11—K1—C2	−127.2 (4)
C7—O4—K1—K1^i^	−104.6 (4)	C12—C11—K1—C2	3.0 (4)
C6—O4—K1—K1^i^	37.5 (3)	O6—C11—K1—C3	−135.9 (4)
C8—O5—K1—O1	−152.3 (3)	C12—C11—K1—C3	−5.7 (5)
C9—O5—K1—O1	−24.1 (4)	O6—C11—K1—I3	−49.8 (4)
C8—O5—K1—O4	17.0 (3)	C12—C11—K1—I3	80.4 (4)
C9—O5—K1—O4	145.2 (4)	O6—C11—K1—K1^i^	137.9 (3)
C8—O5—K1—O6	−150.3 (4)	C12—C11—K1—K1^i^	−91.9 (4)
C9—O5—K1—O6	−22.1 (3)	C4—C3—K1—O1	−148.6 (6)
C8—O5—K1—O3	30.6 (4)	O2—C3—K1—O1	−16.2 (5)
C9—O5—K1—O3	158.8 (3)	C4—C3—K1—O4	40.3 (6)
C8—O5—K1—O3^i^	116.9 (4)	O2—C3—K1—O4	172.6 (5)
C9—O5—K1—O3^i^	−114.9 (3)	C4—C3—K1—O5	62.4 (9)
C8—O5—K1—C2	−109.6 (5)	O2—C3—K1—O5	−165.2 (3)
C9—O5—K1—C2	18.6 (6)	C4—C3—K1—O6	−169.8 (5)
C8—O5—K1—C11	−163.1 (4)	O2—C3—K1—O6	−37.4 (6)
C9—O5—K1—C11	−34.9 (3)	C4—C3—K1—O2	−132.4 (10)
C8—O5—K1—C3	−8.4 (6)	C4—C3—K1—O3	8.2 (5)
C9—O5—K1—C3	119.8 (5)	O2—C3—K1—O3	140.6 (6)
C8—O5—K1—I3	−58.8 (3)	C4—C3—K1—O3^i^	−59.8 (6)
C9—O5—K1—I3	69.4 (3)	O2—C3—K1—O3^i^	72.6 (5)
C8—O5—K1—K1^i^	78.6 (4)	C4—C3—K1—C2	−161.4 (8)
C9—O5—K1—K1^i^	−153.3 (3)	O2—C3—K1—C2	−29.0 (4)
C11—O6—K1—O1	33.9 (4)	C4—C3—K1—C11	−147.6 (6)
C10—O6—K1—O1	165.0 (4)	O2—C3—K1—C11	−15.3 (6)
C11—O6—K1—O4	−156.9 (4)	C4—C3—K1—I3	113.6 (6)
C10—O6—K1—O4	−25.9 (4)	O2—C3—K1—I3	−114.0 (5)
C11—O6—K1—O5	−144.0 (4)	C4—C3—K1—K1^i^	−33.3 (6)
C10—O6—K1—O5	−12.9 (4)	O2—C3—K1—K1^i^	99.0 (5)
C11—O6—K1—O2	43.7 (4)	I2—Cd1—I1—Cd1^ii^	114.57 (2)
C10—O6—K1—O2	174.8 (4)	I3—Cd1—I1—Cd1^ii^	−116.930 (19)
C11—O6—K1—O3^i^	−56.3 (4)	I1^ii^—Cd1—I1—Cd1^ii^	0.0
C10—O6—K1—O3^i^	74.8 (4)	I2—Cd1—I3—K1	55.85 (3)
C11—O6—K1—C2	53.2 (4)	I1—Cd1—I3—K1	−70.15 (3)
C10—O6—K1—C2	−175.8 (4)	I1^ii^—Cd1—I3—K1	−174.64 (2)
C10—O6—K1—C11	131.1 (6)	O1—K1—I3—Cd1	−65.72 (9)
C11—O6—K1—C3	58.5 (5)	O4—K1—I3—Cd1	112.93 (8)
C10—O6—K1—C3	−170.4 (4)	O5—K1—I3—Cd1	174.47 (8)
C11—O6—K1—I3	131.9 (4)	O6—K1—I3—Cd1	−124.39 (9)
C10—O6—K1—I3	−97.0 (4)	O2—K1—I3—Cd1	−5.86 (8)
C11—O6—K1—K1^i^	−54.7 (4)	O3—K1—I3—Cd1	54.07 (8)
C10—O6—K1—K1^i^	76.4 (4)	C2—K1—I3—Cd1	−26.22 (15)
C3—O2—K1—O1	161.4 (5)	C11—K1—I3—Cd1	−107.96 (11)
C2—O2—K1—O1	34.7 (5)	C3—K1—I3—Cd1	13.90 (13)
C2'—O2—K1—O1	−0.7 (18)	K1^i^—K1—I3—Cd1	62.41 (7)

Symmetry codes: (i) −*x*+1, −*y*, −*z*+1; (ii) −*x*, −*y*, −*z*.
